# Effectiveness of a program to lower unwanted media screens among 2–5-year-old children: a randomized controlled trial

**DOI:** 10.3389/fpubh.2024.1304861

**Published:** 2024-06-18

**Authors:** Nimran Kaur, Madhu Gupta, Venkatesan Chakrapani, Firoz Khan, Prahbhjot Malhi, Tanvi Kiran, Sandeep Grover

**Affiliations:** ^1^Department of Community Medicine and School of Public Health, Postgraduate Institute of Medical Education and Research (PGIMER), Chandigarh, India; ^2^Centre for Sexuality and Health Research and Policy (C-SHaRP), Chennai, India; ^3^Department of Pediatrics, PGIMER, Chandigarh, India; ^4^Department of Psychiatry, PGIMER, Chandigarh, India

**Keywords:** screen time, digital media, unwanted media screen, effectiveness, RCT

## Abstract

**Background:**

Limited interventions exist on reducing unwanted screen time (ST) among children from low- and middle-income countries (LMICs), so we developed and assessed the effectiveness of the program to lower unwanted media screen time (PLUMS) among children aged 2–5 years in Chandigarh, Union Territory, North India.

**Methods:**

An open-label randomized control parallel group trial per CONSORT guidelines was conducted among randomly selected 340 families with children aged 2–5 (±3 months) years in Chandigarh, India. PLUMS was implemented at the family level with a focus on modifying the home media environment and targeted individual-level interventions using parent and child modules for 2 months. A post-intervention (immediately) and a follow-up assessment after 6 months was done. During the follow-up period, the interaction was done passively via WhatsApp groups. The control group received routine healthcare services. Validated and standardized tools, including a digital screen exposure questionnaire with a physical activity component, preschool child behavior checklist, and sleep disturbance scale for children, were used to collect data at baseline, post-intervention, and follow-up periods. The primary outcome was the mean difference in ST (minutes/day) among children in the intervention group versus the control group. Generalized estimating equation (GEE) analysis was performed to adjust for clustering.

**Results:**

An equal number of families (*n* = 170) were randomly assigned to the intervention and control arms. In the post-intervention assessment, 161 and 166 families continued while, at the follow-up assessment, 154 and 147 were in the intervention and control arm, respectively. The mean difference in ST on a typical day [27.7 min, 95% Confidence Interval (CI) 5.1, 50.3] at the post-intervention assessment significantly (*p* < 0.05) decreased in the intervention (102.6 ± 98.5 min) arm as compared with the control (130.3 ± 112.8 min) arm. A significant reduction in ST (*β* = −35.81 min, CI -70.6, −1.04) from baseline (*β* = 123.1 min) to follow-up phase (*β* = 116 min) was observed in GEE analysis. The duration of physical activity increased both at post-intervention (*β* = 48.4 min, CI = +6.6, +90.3) and follow-up (*β* = 73.4 min, CI = 36.2, 110.5) assessments in the intervention arm.

**Conclusion:**

The PLUMS intervention significantly reduced the children’s mean ST on a typical day and increased the physical activity immediately post-intervention and during the 6-month follow-up period. These results might guide the policymakers to include strategies in the national child health programs in the Southeast Asia Region to reduce unwanted ST.

**Clinical trial registration**: https://clinicaltrials.gov/, identifier CTRI/2017/09/009761.

## Introduction

Excessive Screen Time (ST) among young children is a significant public health problem globally, with implications for their growth and development (1). ST represents an individual’s use of electronic devices (2) with or without the Internet. Since the 70s, the age at which children begin interacting with the electronic devices has shifted from the older children (4 years old) to the younger ones (4 months old), meaning they are born in a “dynamic digital ecosystem” (3). The early childhood phase (birth to 5 years) is crucial for instilling healthy habits of minimal sedentary screen-based behaviors for optimal health (4).

According to the Indian (2020) (5) and American (2016) (6) Academy of Pediatrics guidelines, a ST of more than 1 h per day in children aged 2–5 years is considered excessive. However, approximately six in ten Indian children aged 2–5 years exceed the daily ST which is permitted (1 h per day) by the age-specific guidelines (7). There are early (delayed motor skills, cognitive and language development, reduced sleep, and disrupted nighttime sleep) and late (prevalence of overweight, obesity, and NCDs) health consequences of excessive ST among children (1, 8). Kaur et al. reported a 59.5% prevalence of exaggerated ST among children aged 2 to 5 years in Chandigarh, India. Excessive ST was found to be significantly associated with emotional problems (15%), sleep problems (8%), and physical inactivity (46.5%) (7).

Nevertheless, researchers acknowledge the benefits of high-quality preschool programs for improving learning outcomes, enhancing literacy skills, and developing vocabulary and comprehension with interactive media, especially during pandemic situations such as the COVID-19 pandemic that had significantly increased early childhood learning (9, 10). Preschoolers play digital games and attach meanings to them, and if used meaningfully, it could improve their learning abilities (11). Most learning apps positively affect the child’s computational thinking skills and classroom learning (12). There are lacunae concerning learning-based interventions for children aged 2–5 years in India to effectively manage media exposure and its resultant ill effects (short- and long-term effects). Since most learning apps positively affect the child’s computational thinking skills and learning (9), the current study used an experimental approach to provide a feasible delivery of offline and online training interventions.

A narrative review by Kaur et al. (1), where intervention studies were reviewed, reported a significant reduction in ST by effective intervention strategies such as increasing digital-media literacy of the parents, reducing sedentary time, controlling the duration of ST, restricting use of electronic devices to age-specific content, family-based counseling, and excessive eating (1, 13–15). They reported that almost all the intervention studies to reduce ST among children younger than years were conducted in developed countries, and seven out of seventeen of the studies concentrated primarily on ST reduction (1, 16–22). The reduction in ST among children of younger than 5 years varied from 0.3 (SE = 13.3) min to 47.16 (SE = 2.01) min in high-income countries. However, no such studies were from middle- and low-income countries (1).

Previous Asian studies on ST aimed to study Internet usage among older children. According to Pedersen et al., recreational intervention to reduce ST resulted in a sizable increase in children’s involvement in physical activity (13, 23). Kaur et al. have reported a significant association of excessive ST with sleep and emotional behaviors (7, 8). Limited interventions were done to reduce ST in children in low- and middle-income countries (LMICs), especially in lowering ST at preschool age, which is a critical developmental stage of learning in school and at home (1). Recently, Poonia et al. reported an RCT in Delhi, India, where parental education starting in infancy in the clinic-based setting (immunization) had shown a decline in ST among children (24).

After reviewing the gap in the existing literature, a comprehensive Program to Lower Unwanted Media Screens (PLUMS) was developed based on the Socioecological model (25), Social Cognitive model (26, 27), and Self-determination theory with motivational interviewing of the parents (27). As digital-screen exposure usually occurs at home, PLUMS was designed to target the family to change the family’s media literacy and home-media environment and develop parent and child-specific modules to intervene and eventually reduce the ST among children (1). Additionally, as there was evidence that children should be involved in decision-making and goal-setting so that parents could channel their children’s energy and help them gain independence for sustained behavior change, social cognitive theory was used to design the intervention (1).

To the best of our knowledge, this is one of India’s first intervention studies using an indigenously designed parents’ and child’s learning modules to reduce ST among 2–5-year-old children. This study substantially differs from the existing studies as it used an RCT-based approach for evaluating the effectiveness of the intervention in decreasing unwanted ST among this specific pediatric age group. In addition, we used a mixed (online and offline) approach to provide knowledge to the families. The primary objective of the study was to assess the effectiveness of PLUMS in reducing ST among children aged 2–5 years in Chandigarh, Union Territory, North India, and secondary objectives were to evaluate the status of emotional problems, sleep problems, and physical activity among children after the intervention.

## Methodology

This trial was registered in the Clinical Trial Registry India: Clinical Trial Registry India CTRI/2017/09/009761: Available at: https://ctri.nic.in/Clinicaltrials/pmaindet2.php?trialid=20050&EncHid=&userName=CHILDREN.

The study protocol is given in detail elsewhere (28). In brief, the methodology is described here.

### Study design and settings

An open-label randomized controlled parallel-group trial was conducted according to the CONSORT guidelines in Chandigarh, a north Indian Union Territory, from October 2020 to August 2021. The CONSORT checklist is shown in Supplementary Table S1. We used a randomized control trial to address the research questions, as these are one of the best study designs with randomly assigned controls to measure the effectiveness of the intervention and generate robust evidence in this regard (28). The study area was the field practice area of the Department of Community Medicine and School of Public Health, Postgraduate Institute of Medical Education and Research (PGIMER), i.e., zone three, Chandigarh, as demographic surveillance was set up in this area. The total population of this area was nearly 250,000, as per the annual health survey report for 2019–2020. There were 8,681 families with children aged 2–5 (±3 months) years in the study area.

### Study participants

The unit of intervention was a family with a child of 2–5 years old. The intervention was delivered at the family level, and the primary caregiver was selected to provide the intervention. The primary caregiver was the person who spent the maximum time with the child and was involved in childcare decision-making. The eligibility criteria included a family who had consented in writing was a resident of the study area for the past 6 months and intended to stay in the study period till the follow-up period was completed. According to medical records, children previously diagnosed with long-term/chronic illnesses were excluded from the study.

### Sample size

The sample size for individual-level randomization was estimated by using the formula (29); N_I_ = (σ_1_^2^ + σ_2_^2^) (Z_1-α/2_+ Z_1-β_)^2^/Δ^2^ (30), where, n = sample size, σ_1_ = standard deviation of the control group and assumed it to be 2.09 (31), σ_2_ = standard deviation of the intervention group and assumed to be 1.46 (31), Δ = difference in group means of average ST and is considered 0.54 (31), Z_1-α/2_ = two-sided Z value (Z = 1.96 for 95% confidence interval), and Z_1-β_ = power (80%). Hence, N_I_ came out to be 161 per arm. After considering the attrition rate of 5%, the sample size was estimated at 170 participants per arm, i.e., 340 for the intervention study.

### Sampling technique and randomization

**Figure 1:**
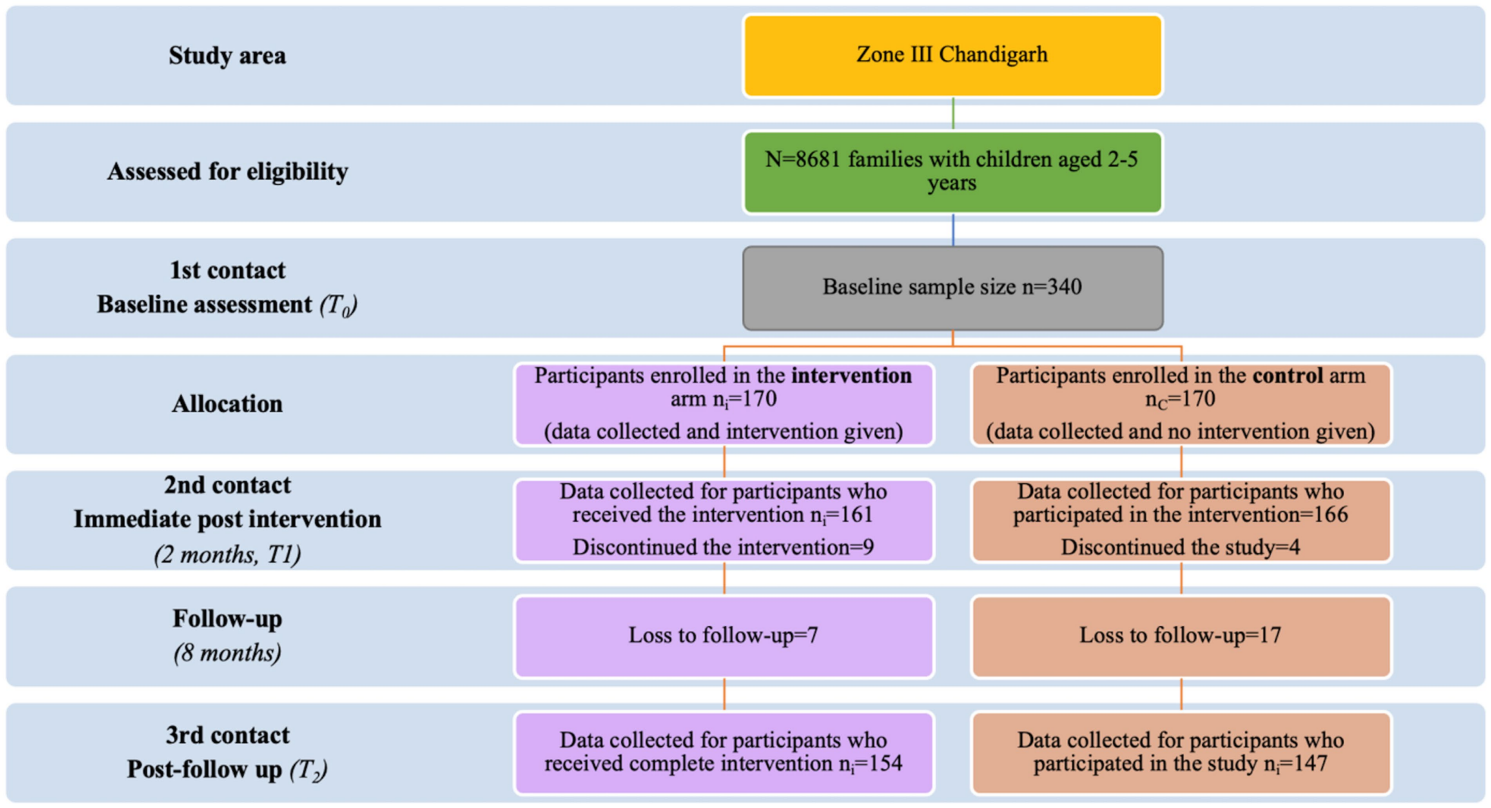
CONSORT flow chart of randomized controlled trial.

A list of eligible families was obtained from the study area’s auxiliary nurse-midwife’s annual health survey register. These families were numbered, and a computer-generated randomized list of the sequences was generated to randomize the families into intervention and control arms to avoid selection bias. The participants were recruited until each arm’s desired sample size was achieved. Each family in the study area had an equal chance of receiving or not receiving the intervention. The study flow diagram, according to the CONSORT guidelines, is shown in Figure 1.

### Concealment of intervention

Th**e** intervention package consisted of information, education, and communication material. The participants were made to understand the intervention to change their media behaviors and reduce ST among children; hence, concealment of the intervention was impossible.

### Blinding

Blinding of the investigator and participants was not possible in this study as the investigator had implemented the intervention herself (first author). The participants were also aware that they were being given the intervention to reduce the ST of their children. This was required as the intervention was targeted for changing the behaviors of the primary caregivers and family members by motivational interviewing methodology. However, data were entered by a blinded data entry operator.

### PLUMS intervention

The intervention program was developed in four phases and was described elsewhere in detail (28). The intervention package is shown in Supplementary File S1. In phase one, an extensive literature review was done to identify the most successful strategies to reduce unwanted ST and understand the theories (such as Social Cognitive Theory for changing children’s behavior and Self-determination theory for caregivers) (1).

The Social Cognitive Theory was used to modify preschoolers’ cognitive development by developing targeted strategies to modify behaviors of the caregivers/children regarding ST or home media environment. When designing the intervention, we used this theory’s framework for modeling (by the caregivers and healthcare worker), production (alternatives to ST, such as activities given to children or suggested by them), retention (repeated positive feedback provided by a healthcare worker), and reinforcement (rewards for encouraging positive behavioral outcomes offered by the healthcare worker) in observational learning, which is pivotal due to the limited cognitive development of preschoolers (26). In addition, self-determination theory was used to design the strategies to keep the caregivers motivated to limit the ST among the children and modify the home media environment. Self-determination theory targets three primary needs: competence, autonomy, and internalization of the learned concepts leading to desired outcomes (27).

According to a systematic review, the most effective treatments for reducing ST in children (0–5 years) were those that lasted for more than or equal to 6 months and were delivered in a community setting, which might have helped in bringing about a long-term positive behavior change among the parents and children (32). The intervention plan was adapted to the Indian setting, based on the content on ST reduction for 2–5-year-old children, as recommended by the Centers for Disease Control and the American Academy of Pediatrics’ official websites. Hence, PLUMS was developed as a family-oriented, theme-based intervention based on Social Cognitive Theory and Self-determination theory (28).

The literature review helped us to identify the modifiable risk factors for each level, including a child (demographic, behavioral, and biological factors), caregiver (demographic, behavioral, and biological factors), and home-media environment level (access to electronic devices, digital-media rules, background TV, etc.); hence intervention was designed likewise (Supplementary Figure S1). We adapted the “Social–Ecological Model” so that the PLUMS modules could effectively target the child’s entourage with specific strategies to reduce the unwanted excessive media exposure among children at the micro (child) level and caregiver along with the home-media environment at the meso level. The conceptual model based on the socio-ecological model is shown in Supplementary Figure S2.

In the second formative phase, in-depth interviews with caregivers (*n* = 20) and two focus group discussions with healthcare providers (*n* = 11), including clinicians (pediatricians), community physicians, and psychologists, were conducted to get their views and opinions on the effective intervention strategies to reduce the unwanted ST in the Indian context. In the third phase, we developed context-specific caregiver/parent and child modules in English and Hindi, with targeted interventions including media-free activities for children and videos for parents on how to engage children in these activities. The contents of these modules were discussed in a consultation meeting with the experts, including psychiatrists, psychologists, and public health experts, before finalizing. The details of the modules and intervention packages are described elsewhere (1).

In brief, the parents and child’s module had eight weekly themes. The parent/caregiver had a choice of 10–12 activities per week for children to choose from every day for the specific theme of that week. Week one focused on excessive screen-time and development milestones of children, week two focused on screen-time rules at home, week three focused on sleep and digital media, week four focused on home media environment, week five focused on meal time and digital media gadgets, week six focused on effective communication within family regarding focusing on home media environment, week seven focused on education in positive experiences in a community setting, week eight focused on positive reinforcement and counseling as shown in the weekly calendar in Supplementary File S1. It was envisaged that child would need to spend 30–60 min per activity per day. There were information-based videos for the parents on the same themes to engage the children in activities which were free from media screens. The motivational counseling was given to parents weekly via phone/video calls, in addition to the next week’s theme-based goals. Specifically, those parents who were not actively participating in sharing the videos of activities were selected and counseled on the perceived barriers/inhibitions. These modules were pretested among 10 families in the fourth phase to determine the intervention strategies’ acceptability, feasibility, and compliance. The final child and caregiver/parent modules are shown in Supplementary File S1.

### Study instruments

The study instruments used to measure the study’s outcomes, which were proxy-reported by the caregivers, are as follows:

- Digital-screen exposure questionnaire (DSEQ): This is a pretested and validated tool to assess the mean ST and physical activity (PA) (33). It has 86 items under five domains, including sociodemographic (age, sex, socioeconomic status, parents’ education, occupation, and religion), screen-time exposure and home media environment, level of physical activity, media-related behaviors, and parental perceptions. For screen time estimation, the frequency, duration, and content of media watched by the child on a typical day were recorded using validated digital screen exposure questionnaire (DSEQ). The DSEQ had good internal consistency, reliability (Cronbach’s alpha = 0.73–0.82), and good inter-rater agreement (Kappa = 0.75, 95% CI 0.72–0.78) (7, 28, 33). The physical activity questions were taken from PrePAQ and reported by parents via face-to-face interviews (7, 29, 34). The questionnaire was used to obtain information on ST in children, digital-screen exposure patterns, caregivers’ perceptions of digital-screen exposure, and the child’s physical activity in liaison with the existing literature (11). The time spent on online classes was recorded separately.
- Pre-school Child Behavior Check List: It assesses the child’s emotional and behavioral problems. It has been shown to have high reliability and validity in the Indian setting (Cronbach’s alpha = 0.95) (34). It is 100 items with a 3-step response scale: absent (score, 0), occasionally present (score, 1), and very often present (score, 2).
- Sleep Disturbance Scale for Children: It is a validated tool and was used for measuring the child’s sleep patterns, which has a good consistency (Cronbach’s alpha = 0.71–0.79, test–retest reliability of 0.71, and diagnostic accuracy of 0.91) (35). It is a 26-item Likert-type scale that measures specific sleep problems and overall sleep disturbances in children.

### Data collection procedures

The intervention study was conducted in three phases: baseline, intervention, post-intervention assessment, and follow-up phase. The CONSORT flow chart depicting the enrolment and loss to follow-up at each assessment point in the intervention and control arm is shown in Figure 1. The DSEQ, PA, emotional and behavioral problems, and sleep disturbances were measured by visiting the homes of the eligible families and conducting face-to-face interviews with the primary caregivers at each assessment point.

We trained two post-graduate level (Masters in Social Work and MA Sociology) field investigators in assisting the first author (NK) in collecting the data in the community during baseline and endline assessments. Field investigators also helped in identifying the families in the community as per the sampling plan. NK herself delivered the intervention at household level weekly and also provided videos on WhatsApp to the primary care givers. She had created WhatsApp group with the participants in the intervention arm for monitoring the progress of the intervention weekly and ensuring positive reinforcement. NK actively participated in delivering the intervention as per the weekly plan and conducting motivational interviewing with the primary caregivers at the household level by personally visiting them and also telephonically. Similar WhatsApp group was also created for the control group, but no intervention material was shared with them, and none of the control families visited during intervention phase. MG, SG, and PM (authors) were involved in weekly supervision and validation of the data collection. Overall, 10% of the data collected was validated by them. The WhatsApp group was monitored by them weekly.

#### Baseline assessment phase (T_0_)

All the families with 2–5-year-old children in the study area were assessed for eligibility. The baseline assessment was initially done from December to March 2020 (25) but had to be repeated from 29th October to 21st November 2020 due to the COVID-19 pandemic, which might have influenced the ST at the baseline due to the lockdown. Then, 340 parents were randomly enrolled in the intervention (*n* = 170) and control arm (*n* = 170).

#### Intervention phase (T1)

The intervention was implemented for 2 months (22nd November 2020 to 3rd January 2021) in the intervention arm at the household level, whereas the control arm received regular health services. There were 170 families in each arm at baseline. Due to the lack of follow-up at the post-intervention assessment, there were 161 in the intervention and 166 in the control arm in this phase. The PLUMS intervention continued for 2 months. Weekly videos (2 min) were shared with the parents on a WhatsApp group during the intervention. The first author performed motivational counseling sessions for the parents who were not participating actively or had inhibition about the intervention plan. The motivational counseling was provided via phone/video calls at a convenient time due to COVID-19 travel restrictions. The daily time spent by the families on the activities was 30–60 min per day. Parents were asked to change the home media environment as a part of the intervention (Supplementary File S1). The control group received the routine care by the health system and given the assessments by us.

#### Post-intervention assessment (T1) and follow-up assessment phase (T2)

A post-intervention immediate evaluation (T_1_) was conducted from 11th January 2021 to 31st January 2021 using the same tools as used in baseline assessment at T_0_. Follow-up of the families continued for another 6 months after intervention from 1st February to 13th August 2021. In this phase, 154 families remained in the intervention group and 147 in the control arm. This maintenance phase included sharing information, communication, and educational material fortnightly with the parents on the WhatsApp group, as shown in Supplementary File S1. A follow-up assessment after 6 months of intervention was conducted using the same tools as the baseline assessment conducted in August 2021.

### Outcomes

The primary outcome was the mean differences in ST (in minutes per day) separately on typical days [this was the average of 7 days in a week calculated by deriving the weighted average of weekdays and weekends in a manuscript published elsewhere (7, 8)], weekdays (Monday–Friday), and weekends (Saturday and Sunday) in the intervention versus the control arm. The secondary outcomes of the study were the difference in proportions of children with excessive ST, emotional behavior problems, sleep problems, change in media rules at home, and the duration of the physical activity (in minutes) per day in the intervention versus the control arm.

### Adherence to the intervention

During the weekly counseling sessions, the compliance proforma was given to the caregivers to check their fidelity during the active intervention phase (2 months). The child’s adherence and level of engagement were assessed with the help of activity videos and/or pictures shared by the parents on the WhatsApp group during the 8-week intervention period. The compliance proforma has been published elsewhere (28).

### Data analysis

Data was entered into a Microsoft Excel sheet and analyzed using IBM SPSS for Macintosh version 25.0. and StataCorp. 2019. *Stata Statistical Software: Release 16*. College Station, TX: StataCorp LLC. The results are presented per the Intention to Treat (ITT) analysis. The ITT approach was preferred as it preserves the balance by maintaining the comparability between treatment and control arms, reducing the risk of bias, and maintaining the effect of randomization, thereby retaining the power of the research study. Missing values were excluded from the analysis.

The per-protocol analysis was also executed for exploratory assessments. A *p*-value <0.05 was considered statistically significant for all analyses at a 95% confidence interval (CI). According to Kolmogorov–Smirnov test results, ST data were non-normally distributed for children’s ST (36). The medians, standard deviation (SD), standard error (SE), and interquartile ranges were estimated for continuous variables. The differences in means between the two groups regarding ST of the children on a typical day, weekday, and weekend were assessed by the Mann–Whitney U test (36).

For categorical variables, the proportions were calculated. The differences in proportions between the two groups were tested using the chi-square and Fisher exact tests (for less than five observations). The relative risk reduction (1-relative risk*100) was estimated to assess the effectiveness of the intervention. As this was longitudinal data, the Generalized Estimating Equation approach was used to adjust the effect of clustering on the effectiveness of the intervention. The models were developed for dependent variables, including ST as a continuous variable (average ST of the child in minutes on a typical day), ST as a categorical variable (proportion of children having ST of less than one versus more than 1 h per day), emotional problems (scores), sleep problems (scores), and duration of physical activity (minutes). Independent variables adjusted were the child’s age, sex of the child, socioeconomic status of the family, average daily father’s ST, father’s education, average everyday mother’s ST, mother’s education, and digital media rules.

### Ethical considerations

The Ethics Committee of the Postgraduate Institute of Medical Education and Research, Chandigarh, approved the study (INT/IEC/2019/000711), and all prior permissions were obtained to conduct research in the community setting (VO/FW/17/1894, Dated-30/08/17). Verbal and written informed consent was obtained from the parents before giving the PLUMS intervention.

## Results

The randomization of families is illustrated using the Consolidated Standards of Reporting Trials (CONSORT) flow chart, as shown in Figure 1. This CONSORT flow chart depicts the enrollment and loss to follow-up at each assessment point in the intervention and control arm.

Among the parents in the intervention arm (*n* = 170) who had received the video intervention, approximately 94.7% completed the weekly activities, and 61% required motivational counseling. It can be observed that in 46% of parents, fathers changed their behavior. Most parents acknowledged that the intervention was simple and understandable with easy-to-perform activities. There was an increase in the participants’ knowledge concerning ST (Supplementary Table S2).

There were more or less similar numbers of participants in both the intervention and control arms to the sex and age of the child. Predominantly, the study participants belonged to the Hindu religion and were urban residents. Most of the children belonged to the nuclear family setup, wherein the child’s mother was the primary caregiver. Most of the parents were married, wherein for most participants, the mother’s age was younger than 30 years old, and that of the fathers’ was older than 30 years for both the control and intervention arms. Mothers were slightly more educated in the intervention arm than in the control arm. However, the majority of the mothers were unemployed for both arms. There were no significant differences between the background characteristics of the children in the intervention versus the control arm, and the children were equally distributed among both arms (Table 1). On a typical day, the mean ST of children aged 2–5 years in Chandigarh had a significantly non-normal distribution as represented by Whisker box plots (Supplementary Figure S3).

**Table 1:** Background characteristics of the parents of children aged 2–5 years in Chandigarh in the intervention and control groups in 2021.

Variable	Intervention arm *N* = 170 (%)	Control arm *N* = 170 (%)
Child’s sex		
Boys	86 (50.6)	87 (51.2)
Girls	84 (49.4)	83 (48.8)
Age of the child		
2 to <3 years	47 (46.5)	54 (53.5)
3–4 years	17 (45.9)	20 (54.1)
>4 to 5 years	103 (52.6)	93 (47.4)
Religion		
Hindu	140 (82.4)	149 (87.6)
Sikh	15 (8.8)	12 (7.1)
Muslim	12 (7.1)	9 (5.3)
Others	3 (1.8)	0
Place of residence		
Urban	90 (52.9)	85 (50)
Resettlement colony/urbanized village	80 (47.1)	85 (50)
Family type		
Nuclear	104 (61.2)	115 (67.6)
Extended/joint	66 (38.8)	55 (32.4)
Parents’ marital status		
Married	167 (98.2)	167 (98.2)
Divorced/separated/single	3 (1.8)	3 (1.8)
Mother’s age		
Less than 30 years	103 (60.9)	93 (54.7)
More than 30 years	66 (39.1)	77 (45.3)
Father’s age		
Less than 30 years	56 (32.9)	52 (30.6)
More than 30 years	114 (67.1)	118 (69.4)
Primary caregiver of the child		
Mother	160 (94.1)	159 (93.5)
Father	3 (1.8)	3 (1.8)
Grandfather	3 (1.8)	1 (0.6)
Grandmother	6 (3.5)	4 (2.4)
Others	0	1 (0.6)
Mother’s education		
Illiterate/primary school	29 (17.1)	22 (12.9)
Middle school	25 (14.7)	25 (14.7)
High school intermediate diploma	59 (34.7)	47 (27.6)
Graduation/professional honors	57 (33.5)	75 (44.1)
Father’s education		
Illiterate/primary school	19 (11.2)	18 (10.6)
Middle school	40 (23.5)	31 (18.2)
High school/Intermediate diploma	55 (32.4)	49 (28.8)
Graduation/professional honors	56 (32.9)	72 (42.4)
Per capita income of the family*		
Below Rs.11000 (USD 147)	102 (60)	118 (69.4)
Above Rs.11000	68 (40)	52 (30.6)
Mother’s occupation		
Unemployed	151 (88.8)	140 (82.4)
Employed	19 (11.2)	30 (17.6)
Father’s occupation		
Legislator/senior officer/manager/professionals	34 (20)	42 (24.7)
Technician/associate professional/clerks	29 (17.1)	40 (24.4)
Skilled worker/craft related worker/plant operator	88 (39.4)	83 (48.8)
Unemployed	1 (0.6)	0

*1 USD = 75 INR.

The mean ST (27.7 min, CI = 5.1, 50.3, *p* = 0.011) at the immediate post-intervention assessment significantly decreased in the intervention arm (102.6 ± 98.5 min) as compared with the control arm (130.3 ± 112.8 min) on a typical day. The mean ST of children on the weekend reduced significantly at the follow-up assessment (21.7 min, CI = −4, 47.3, *p* = 0.041) in the intervention arm (105.4 ± 124 min) versus the control arm (127.1 ± 116.5 min). However, there were no significant changes on the weekdays. The duration of physical activity was significantly (*p* < 0.0001) different at the follow-up between the intervention and control arm (Table 2).

**Table 2:** Mean screen time (in minutes) of children aged 2–5 years in Chandigarh on a typical day, weekday, and weekend in the intervention and control arms in 2021.

Variables	Intervention arm (*N* = 170)	Control arm (*N* = 170)	The difference in ST (min)	95% CIs	*P*-value
A. Typical day					
	Mean ST ± SD (SE)	Mean ST ± SD (SE)			
Baseline (T_0_)					
Mean screen time	123.1 ± 83.1 (6.3)	130.5 ± 114.8 (8.8)	7.4	−13.9, 28.8	0.36
Post-intervention (T_1_)	*N* = 170	*N* = 170			
Mean screen time	102.6 ± 98.5 (7.5)	130.3 ± 112.8 (8.6)	27.7	5.1, 50.3	0.011
Follow-up (T_2_)					
Mean screen time	116 ± 114.9 (8.8)	136.6 ± 118.1 (9)	20.7	−4.1, 45.6	0.09
B. Weekday					
Baseline (T_0_)					
Mean screen time	125.9 ± 81 (6.2)	135.6 ± 121.6 (9.3)	9.7	−12.3, 31.7	0.4
Post-intervention (T_1_)					
Mean screen time	113.7 ± 109.3 (8.3)	133.2 ± 120.4 (9.2)	19.5	−5, 44	0.12
Follow-up (T_2_)					
Mean screen time	126.8 ± 130.3 (9.9)	146.1 ± 138.1 (10.6)	19.2	−9.4, 47.9	0.13
C. Weekend					
Baseline (T_0_)					
Mean screen time	120.4 ± 95.5 (7.3)	125.4 ± 116.6 (8.9)	5	−17.8, 27.7	0.5
Post-intervention (T_1_)					
Mean screen time	104 ± 115.2 (8.8)	117.7 ± 121.4 (9.3)	13.7	−11.5, 39	0.19
Follow-up (T_2_)					
Mean screen time	105.4 ± 124 (9.5)	127.1 ± 116.5 (8.9)	21.7	−4, 47.3	**0.041**

*T_0_ is the baseline assessment, T_1_ is the post-intervention assessment, and T_2_ is the follow-up assessment.

The proportion of families in the intervention arm changed the placement of the TV significantly (*p* = 0.04) and increased in the intervention arm at the immediately post-intervention assessment point (*N* = 78, 44.1%), as compared with the control arm (*N* = 99, 59.6) from the baseline assessment point (intervention 56.5%, control 58.8%). The proportion of families in the intervention arm who changed the placement of the smartphone significantly (*p* = 0.01) increased at the immediately post-intervention (intervention *N* = 98, 61.3%; control *N* = 123, 74.5%) and follow-up assessment (intervention *N* = 123, 83.7%, control *N* = 109, 71.2%) from the baseline assessment (intervention *N* = 135, 79.4%, control *N* = 141, 82.9%) points.

The relative risk of having excessive ST among children immediately post-intervention was 0.79. The PLUMS intervention had statistically significant moderate effectiveness in reducing the excessive ST at the immediately post-intervention assessment period [(1–0.79)*100 = 21%, *p* = 0.0038], but it was insignificant at the follow-up assessment point [(1–0.91)*100 = 9%, *p* = 0.43].

The results of the generalized estimating equation have shown that there was a significant (*p* = 0.04) reduction of ST (*β* = −35.81 min, CI = −70.6, −1.04) in the intervention arm as compared with the control arm from the baseline (T_0_) to the follow-up (T_2_) assessment point. When ST was dichotomized as a categorical variable (less than versus more than 1 h per day ST), there was a significant reduction (adjusted odds ratio = 0.48, CI = 0.15, 0.27) in the proportion of children with excessive ST from baseline (T_0_) to post-intervention (T_1_) assessment points in the intervention as compared with the control arm. The physical activity duration had increased considerably in the intervention arm versus the control arm at both the immediate post-intervention T_1_ (*β* = 48.4 min, CI = +6.6, +90.3) and follow-up T_2_ (*β* = 73.4, CI = 36.2, 110.5) assessment points in the intervention arm as compared with the control arm (Table 3). No significant changes were observed in the children’s sleep and emotional behaviors (Supplementary Table S3).

**Table 3:** Generalized estimating equations for estimating the longitudinal effect of the PLUMS intervention.

Parameters in the intervention versus control arm	Measure	Standard error	95% CI		
			Lower	Upper	*p*-value
Screen time in minutes	Beta coefficient (β)				
Post intervention period (T1)	−13.8	14.8	−42.96	15.31	0.21
Post 6 months (T2)	−35.8	17.74	−70.59	−1.04	0.04
Physical activity in minutes					
Post intervention period (T1)	48.4	21	6.6	90.33	0.023
Post 6 months (T2)	73.37	18.97	36.19	110.55	<0.0001
Emotional problems scores					
Post intervention period (T1)	2.67	1.64	−0.55	5.9	0.1
Post 6 months (T2)	2.06	2.17	−2.18	6.31	0.34
Sleep problem scores					
Post intervention period (T1)	0.86	1.3	−1.84	3.57	0.53
Post 6 months (T2)	1.56	1.41	−1.21	4.33	0.27
Screen time as categorical variable	Adjusted odd’s ratio		Lower CI	Upper CI	*p*-value
Post intervention period (T1)	0.48	0.15	0.27	0.87	0.016
Post 6 months (T2)	0.51	0.18	0.25	1.01	0.056

The model was adjusted for father’s ST and education, mother’s ST and education, digital media rules, area of residence socioeconomic status, and gender and age of the child. ^$^ST, Screen time.

## Discussion

The PLUMS intervention’s focus on enhancing the parents’ ST literacy home-media environment and customizing the counseling sessions seemed to have eventually brought a sustainable change in ST behaviors among Indian children aged 2–5 years. As parents act as an essential liaison between the young child and the health worker, family-based counseling (6) has been proven productive in bringing about behavior change; thus, we incorporated the parents in our intervention for role-modeling as per the socioecological model (25). Additionally, Bandura’s “Social Learning Theory” (37) was incorporated to explain how learning occurs in a social context with a reciprocal and dynamic interaction between the person (here, child/ caregiver), behavior, and environment. As the parents were role-modeling the appropriate behaviors, we used the self-determination theory (38). High-quality learning and favorable outcomes have been observed with the self-determination theory and motivational interviewing (27) when used together.

The PLUMS intervention significantly decreased the mean ST (27.7 min, CI = 5.1, 50.3) at the post-intervention assessment in the intervention arm compared with the control arm on a typical day. Additionally, it effectively reduced the ST (*β* = −35.81 min CI = −70.6, −1.04) among children in the intervention versus the control arm from the baseline to the follow-up assessment points. The duration of physical activity had increased significantly in the intervention arm versus the control arm at both the post-intervention (*β* = 48.4 min, CI = +6.6, +90.3) and follow-up (*β* = 73.4 min, CI = 36.2, 110.5) assessment points. However, sleep problems and emotional problems among children did not change.

Overall, the mean reduction in ST was well within the range [0.3 (SE = 13.3) to −47.16 (SE = 2.01) min], as reported in a review that included 16 intervention studies among children of younger than 5 years old in high-income countries (1). A greater and significant reduction in ST from baseline to follow-up was observed in this study compared with a European intervention study conducted by Yilmaz et al. among 2–6-year-old children (39). Our study followed the latest ST guidelines of less than 1 h per day to determine the ST as excessive. In contrast, previous studies followed older ST guidelines of less than 2 h per day as the permissible ST limit for 2–5-year-old children. The average ST was significantly reduced on a typical day and the weekends but not on the weekdays between the intervention and control groups in this study. Paradoxically, a Canadian study reported no significant change among preschoolers on weekdays and weekends (17). These differences might be due to differences in the study objectives and settings; additionally, we performed home visits (first visit) and gave motivational counseling via phone/video calls. There was a significant change in the placement of electronic devices in the room where the child slept, such as television (*p* = 0.042), smartphone (*p* = 0.01) post-intervention assessment, and only smartphone (*p* = 0.01) at follow-up assessment, as reported in other studies (40, 41).

Our study results observed an increase in the duration of physical activity in children with an improvement in ST literacy. It has been observed in the existing literature that multicomponent intervention studies combining physical activity with nutritional interventions have shown beneficial effects (42). Moreover, it is likely that health education-focused, customized family-based counseling (28) with persistently motivating the participants might bring about sustainable behavior change (1). A 14-week intervention among 18 American schools observed more rigorous physical activity than the children in the control schools (43). The COVID-19 pandemic restrictions might have affected the type of physical activity performed by the children in our study.

The effect of ST on psychological health and cognitive abilities in young children has been previously debated but not investigated in a randomized design, raising questions regarding its causal impact. To reduce ST among children of 4–6 years of age (23), a recent Taiwanese intervention study had observed a significant change in the psychosocial health of children, in contrast to this research. This change can be due to different study settings (classroom or home-based) where appropriate psychosocial behaviors were observed. The Taiwanese study selected children with more than 2 h of ST per day, while we randomly selected children from the community. The PLUMS intervention did not affect sleep duration, sleep patterns, and emotional problems. This could be because the primary focus of this study was reducing sedentary screen behaviors.

However, parents reported a change in their child’s behavior (emotional, physical activity, and sleep patterns) during the COVID-19 pandemic. Another long-term study showed no effect on sleep duration and cognitive abilities among European preschoolers (*n* = 652) (42), which is similar to this study. In contrast, in an American trial among 2–5-year-old children, an increase in the sleep duration (0.56 h per day) in the intervention group and a decrease in the control group (0.19 h per day) were reported (42). In the former study, academicians and health educators gave motivational coaching during four home visits, mailed educational materials, and offered incentives to the study participants, in contrast to the present study. Pediatricians, child psychologists, and educators can devise similar educational modules to counsel families regarding ST, its regulation or alternatives, and its consequences in future studies. Thus, the educational material developed in the current study might also aid teachers, educators, and practitioners in reducing ST among children.

This research is valuable to academicians and researchers as it provides evidence of the effectiveness of PLUMS at the home level in reducing unwanted STs among young children. In the future, they can plan a study on its impact on emotional and sleep behaviors to assess health impacts. For the practitioners, the results of this study provided evidence that motivational coaching of the parents regarding reducing unwanted ST and modifying the home media environment during visits of the children in the clinics can reduce unwanted ST. The practitioners should be sensitized to exploring the digital ST of the young children during such visits.

The strengths of the study included a robust study design, i.e., a randomized control trial among the parents in the community settings conducted for the first time in India among preschoolers to reduce excessive ST. Another strength is the multiphasic development of the PLUMS, which helped identify the problems and related solutions before the implementation. To overcome the recall bias in reporting ST, the first author recorded the names of TV/online programs and the approximate duration the child watched in the past week to calculate the ST accurately. TV diaries were also proposed to the primary caregivers to capture the children’s actual ED use. However, the parents found it challenging to maintain them; instead, they shared a daily activity journal that verified the children’s adherence to the intervention and activities at home. A meta-analysis concluded that interventions enabled the participants to change their behaviors by controlling the electronic devices at home, setting goals, and planning media use (where children participated in this decision-making); children were allowed to watch electronic devices for a specified time, rewards for good behavior, increasing ST literacy or parents. All these strategies were incorporated into the present study. We observed a small effect size (21%) in the post-intervention assessment, supported by the former meta-analysis that ST-focused interventions generate a small effect size due to wider CIs and ranges of participants’ ST, small sample size, which does not affect the overall impact of the intervention (44). The study findings can be generalized to similar urban settings in North India and Southeast Asia.

The study has several limitations. First, the parents worked from home during the COVID-19 pandemic, making it difficult to focus on the child. Moreover, the PLUMS module suggested home-based feasible activities with adult supervision. There was a loss in follow-up in the intervention and control arms (post-intervention-T_1_:4%; follow-up-T_2_:12%), which might be due to the overlapping information of PLUMS with the ongoing online classes for the children. Second, the social desirability and recall bias might have affected some responses reported by the parents. The COVID-19 pandemic might have weakened the impact of the intervention on the emotional and sleep behaviors of the children. Third, fathers’ involvement was low as observed previously that fathers’ participation and retention in an intervention program is challenging in LMICs (45). The only solution to this problem is to customize an intervention plan to make the program feasible and accessible to the participants. Following up with the parents on the phone and sending them brochures on the adverse effects of excessive ST helped increase their participation. Finally, blinding could not be done as this project was a part of the first author’s Ph.D. program, and she gave the intervention to the participants. In addition, the intervention group was performing the activities; hence, they knew they were a part of the intervention. Finally, the study protocol assumed the non-response rate/refusal rate to be 10% and loss to follow-up to be 15%, which estimated the sample size to be 214 per arm (28). However, the sample size estimation was revised after obtaining the Doctoral Committee’s approval due to the loss of participants during the COVID-19 pandemic in the follow-up and the paucity of funds. Moreover, the attrition rate was reduced to 5% to reduce the final sample size (170 per arm). We acknowledge that the intervention aimed to reduce the ST, not necessarily modifying the choice of ST programs/content the children viewed. We also realize that as children’s daily non-sleep time is limited, adding additional activity time exposure to media screen time might decrease naturally, but evidence needed to be generated in this regard.

The study findings have important public health implications. This study has provided evidence that carefully designed; evidence-based, culturally appropriate ST reduction programs can successfully modify the home-media environment and reduce the burden of excessive ST among young children. Hence, home-based intervention programs are potentially needed to decrease the burden of ST and its associates. This study might help implement ST reduction interventions in India and other countries in the Southeast Asian region.

## Conclusion

Based on Social Cognitive and Self-determination theory, an evidence-based, culturally appropriate multicomponent home-based intervention, known as Program to Lower Unwanted Media Screen (PLUMS), was developed and implemented at the micro (child) and meso (family and home environment) level as per the socioecological model. It effectively reduced excessive ST at the child and the family levels. This is one of the first intervention studies from the Southeast Asia Region in this regard. The new knowledge added to the existing literature is that it is feasible to implement PLUMS intervention at the family level in urban Indian settings to effectively reduce unwanted ST among young children, with evidence of its sustainability for 6 months.

### What is known about the subject

Excessive screen-time among children is a significant public health problem globally, with implications for their growth and development. Unwanted screen-time can be effectively reduced; however, almost all intervention studies were conducted in developed countries to mitigate screen-time among children of younger than 5 years old.

### What this study adds

This is the first randomized controlled trial in Indian households to generate evidence on the program's effectiveness in lowering unwanted screens (PLUMS), significantly reducing it among children aged 2–5 years. PLUMS was especially designed to consider the context of low-middle income countries using social cognitive theory for children and self-determination theory for caregivers.

## Data availability statement

The original contributions presented in the study are included in the article/Supplementary material, further inquiries can be directed to the corresponding author.

## Ethics statement

The study involve humans were approved by PGIMER Institutional Ethics Committee, Chandigarh India. (INT/IEC/2019/00711 dated 02/04/2019). The studies were conducted in accordance with the local legislation and institutional requirements. Written informed consent for participation in this study was provided by the participants’ legal guardians/next of kin.

## Author contributions

NK: Conceptualization, Data curation, Formal analysis, Funding acquisition, Investigation, Methodology, Project administration, Resources, Software, Supervision, Validation, Visualization, Writing – original draft, Writing – review & editing. MG: Conceptualization, Funding acquisition, Methodology, Project administration, Resources, Supervision, Validation, Visualization, Writing – review & editing. VC: Data curation, Formal analysis, Methodology, Software, Validation, Visualization, Writing – review & editing. FK: Data curation, Formal analysis, Software, Writing – review & editing, Validation, Visualization. PM: Writing – review & editing. TK: Supervision, Writing – review & editing, Data curation, Formal analysis, Software, Writing – original draft. SG: Conceptualization, Investigation, Software, Supervision, Validation, Visualization, Writing – review & editing.
