# Supplementary material for: Effectiveness of a program to lower unwanted media screens among 2–5-year-old children: a randomized controlled trial
Source: Front Public Health. 2024 Jun 18;12:1304861. doi: 10.3389/fpubh.2024.1304861 (PMC11223730; doi:10.3389/fpubh.2024.1304861)
Supplement: Supplementary file 1 [file Table_1.docx]

Supplementary Table 1: CONSORT 2010 checklist when reporting a randomised trial.

| Section/Topic | Item No | Checklist item | Reported on page No |
| --- | --- | --- | --- |
| Title and abstract | | | |
|  | 1a | Identification as a randomised trial in the title | 3 |
|  | 1b | Structured summary of trial design, methods, results, and conclusions (for specific guidance see CONSORT for abstracts) | 3-4 |
| Introduction | | | |
| Background and objectives | 2a | Scientific background and explanation of rationale | 5 |
|  | 2b | Specific objectives or hypotheses | 6 |
| Methods | | | |
| Trial design | 3a | Description of trial design (such as parallel, factorial) including allocation ratio | 6 |
|  | 3b | Important changes to methods after trial commencement (such as eligibility criteria), with reasons | Not done |
| Participants | 4a | Eligibility criteria for participants | 7 |
|  | 4b | Settings and locations where the data were collected | 6 |
| Interventions | 5 | The interventions for each group with sufficient details to allow replication, including how and when they were actually administered | 7 |
| Outcomes | 6a | Completely defined pre-specified primary and secondary outcome measures, including how and when they were assessed | 10 |
|  | 6b | Any changes to trial outcomes after the trial commenced, with reasons | Not done |
| Sample size | 7a | How sample size was determined | 8 |
|  | 7b | When applicable, explanation of any interim analyses and stopping guidelines | Done |
| Randomisation: |  |  |  |
| Sequence generation | 8a | Method used to generate the random allocation sequence | 6 |
|  | 8b | Type of randomisation; details of any restriction (such as blocking and block size) | 6 |
| Allocation concealment mechanism | 9 | Mechanism used to implement the random allocation sequence (such as sequentially numbered containers), describing any steps taken to conceal the sequence until interventions were assigned | 6 |
| Implementation | 10 | Who generated the random allocation sequence, who enrolled participants, and who assigned participants to interventions | 6 |
| Blinding | 11a | If done, who was blinded after assignment to interventions (for example, participants, care providers, those assessing outcomes) and how | 6 |
|  | 11b | If relevant, description of the similarity of interventions | - |
| Statistical methods | 12a | Statistical methods used to compare groups for primary and secondary outcomes | 8 |
|  | 12b | Methods for additional analyses, such as subgroup analyses and adjusted analyses | 8 |
| Results | | | |
| Participant flow (a diagram is strongly recommended) | 13a | For each group, the numbers of participants who were randomly assigned, received intended treatment, and were analysed for the primary outcome | Published Protocol  Figure 1 |
|  | 13b | For each group, losses and exclusions after randomisation, together with reasons | Figure 1 |
| Recruitment | 14a | Dates defining the periods of recruitment and follow-up | 11 [Figure 1] |
|  | 14b | Why the trial ended or was stopped | 11 |
| Baseline data | 15 | A table showing baseline demographic and clinical characteristics for each group | Table 1 |
| Numbers analysed | 16 | For each group, number of participants (denominator) included in each analysis and whether the analysis was by original assigned groups | 8 |
| Outcomes and estimation | 17a | For each primary and secondary outcome, results for each group, and the estimated effect size and its precision (such as 95% confidence interval) | 8 |
|  | 17b | For binary outcomes, presentation of both absolute and relative effect sizes is recommended | 13 |
| Ancillary analyses | 18 | Results of any other analyses performed, including subgroup analyses and adjusted analyses, distinguishing pre-specified from exploratory | 11-13 |
| Harms | 19 | All important harms or unintended effects in each group (for specific guidance see CONSORT for harms) | - |
| Discussion | | | |
| Limitations | 20 | Trial limitations, addressing sources of potential bias, imprecision, and, if relevant, multiplicity of analyses | 17 |
| Generalisability | 21 | Generalisability (external validity, applicability) of the trial findings | 17 |
| Interpretation | 22 | Interpretation consistent with results, balancing benefits and harms, and considering other relevant evidence | 13-18 |
| Other information | | |  |
| Registration | 23 | Registration number and name of trial registry | Clinical Trial Registry-India (CTRI/2017/09/009761). |
| Protocol | 24 | Where the full trial protocol can be accessed, if available | Kaur N, Gupta M, Malhi P, Grover S  A Multicomponent Intervention to Reduce Screen Time Among Children Aged 2-5 Years in Chandigarh, North India: Protocol for a Randomized Controlled Trial  JMIR Res Protoc 2021;10(2):e24106  doi: [10.2196/24106](https://doi.org/10.2196/24106)PMID: [33570499](https://www.ncbi.nlm.nih.gov/pubmed/33570499)PMCID: [7906833](https://www.ncbi.nlm.nih.gov/pmc/articles/7906833) |
| Funding | 25 | Sources of funding and other support (such as supply of drugs), role of funders | Indian Council of Medical Research, New Delhi (3/1/3/Next-100/JRF-2015/HRD), and intra-mural project funds from PGIMER, Chandigarh (NK/3442/Ph.D/186, Dated 09/05/2017). |

Supplementary Table 2: Feedback of the PLUMS implementation by the parents

| Indicators | Feedback by the participants N (%) |
| --- | --- |
| Family who received the video intervention | 170/170 (100) |
| Parents who completed the activities | 161/170 (94.7) |
| Parents who required motivational counselling | 98/161 (60.9) |
| Both the parents performed the activities with the children | 74/161 (46) |
| Parents who acknowledged that the language of the intervention was simple | 102/161 (63.4) |
| Parents who acknowledged that the activities were feasible and easy to perform | 111/161 (68.9) |
| Parents agreed the action plan was comprehensible or logical or well-styled or correct | 116/161 (72) |
| Proportion of parents’ who were aware of harmful effects of excessive screen time among children (pre- versus post-intervention) | Pre-intervention: 93/170 (54.7)  post-intervention: 120/161 (74.4) |
| Proportion of parents’ who are aware of useful effects of excessive screen time among children (pre- versus post-intervention) | Pre-intervention: 111/170 (34.7)  post-intervention: 124/161 (23.2) |
| Proportion of parents who participated in the weekly video sessions | 161/170 (94.7) |
| Proportion of parents who filled the feedback proforma | 161/170 (94.7) |

**Supplementary Table 3: Comparison of proportion of children’s ST, digital media rules at home, sleep problems, emotional problems and duration of physical activity among children at baseline, post-intervention, and follow-up in Chandigarh 2021**

| **Assessment points** | **Intervention arm, N=170** | **Control arm, N=170** | **P-value** |
| --- | --- | --- | --- |
| 1. ***Digital media rules at home*** |  |  |  |
| **Baseline (T_0_)** |  |  | 0.29 |
| Digital media rules present | 30 (17.6) | 23 (13.5) |  |
| Digital media rules absent | 140 (82.3) | 147 (86.5) |  |
| **Post-intervention (T_1_)** |  |  | 0.43 |
| Digital media rules present | 21 (12.4) | 26 (15.3) |  |
| Digital media rules absent | 149 (87.6) | 144 (84.7) |  |
| **Follow-up (T_2_)** |  |  | 0.47 |
| Digital media rules present | 32 (18.8) | 27 (15.9) |  |
| Digital media rules absent | 138 (81.2) | 143 (84.1) |  |
| 1. ***Sleep problems*** |  |  |  |
| **Baseline (T_0_)** |  |  | 0.19 |
| Normal | 135 (51.1) | 129 (48.9) |  |
| Borderline cases | 20 (11.8) | 25 (14.7) |  |
| Clinical cases | 15 (8.8) | 16 (9.4) |  |
| **Post-intervention (T_1_)** |  |  | 0.96 |
| Normal | 131 (77.1) | 129 (75.9) |  |
| Borderline cases | 28 (16.5) | 30 (17.6) |  |
| Clinical cases | 11 (6.5) | 11 (6.5) |  |
| **Follow-up (T_2_)** |  |  | 0.9 |
| Normal | 128 (75.3) | 125 (73.5) |  |
| Borderline cases | 31 (18.2) | 32 (18.8) |  |
| Clinical cases | 11 (6.5) | 13 (8.8) |  |
| 1. ***Emotional problems*** |  |  |  |
| **Baseline (T_0_)** |  |  | 0.91 |
| Normal | 129 (49.4) | 132 (50.6) |  |
| Borderline cases | 12 (7.1) | 12 (7.1) |  |
| Clinical cases | 29 (17.1) | 26 (15.3) |  |
| **Post-intervention (T_1_)** |  |  | 0.83 |
| Normal | 131 (77.1) | 127 (74.7) |  |
| Borderline cases | 25 (15.3) | 26 (14.7) |  |
| Clinical cases | 14 (8.2) | 17 (10) |  |
| **Follow-up (T_2_)** |  |  | 0.93 |
| Normal | 128 (75.3) | 129 (75.9) |  |
| Borderline cases | 24 (14.1) | 25 (14.7) |  |
| Clinical cases | 18 (10.6) | 16 (9.4) |  |
| 1. ***Duration of physical activity*** |  |  |  |
| **Baseline (T_0_)** |  |  | 0.14 |
| No activity | 56 (32.9) | 71 (41.8) |  |
| Less than 30 min | 114 (67.1) | 98 (57.6) |  |
| More than 30 min | 0 | 1 (0.6) |  |
| **post-intervention (T_1_)** |  |  | 0.43 |
| No activity | 34 (20) | 40 (23.5) |  |
| Less than 30 min | 93 (54.7) | 81 (47.6) |  |
| More than 30 min | 43 (25.3) | 49 (28.8) |  |
| **Follow-up (T_2_)** |  |  | **<0.0001** |
| No activity | 28 (16.5) | 57 (33.5) |  |
| Less than 30 min | 82 (48.2) | 36 (21.22) |  |
| More than 30 min | 60 (35.3) | 77 (45.3) |  |
| Screen time (categorical) |  |  |  |
| **Baseline (T_0_)** |  |  | **0.001** |
| Children having average screen time >1 hour/day | 133 (78.2) | 105 (61.8) |  |
| Children having average screen time <1 hour/day | 37 (21.8) | 65 (38.2) |  |
| **Post-intervention (T_1_)** |  |  | **0.038** |
| Children having average screen time >1 hour/day | 86 (50.6) | 105 (61.8) |  |
| Children having average screen time <1 hour/day | 84 (49.4) | 65 (38.2) |  |
| **Follow-up (T_2_)** |  |  | 0.43 |
| Children having average screen time >1 hour/day | 104 (61.2) | 111 (65.3) |  |
| Children having average screen time <1 hour/day | 66 (38.8) | 59 (34.7) |  |
