# Supplementary material for: Effectiveness of a program to lower unwanted media screens among 2–5-year-old children: a randomized controlled trial
Source: Front Public Health. 2024 Jun 18;12:1304861. doi: 10.3389/fpubh.2024.1304861 (PMC11223730; doi:10.3389/fpubh.2024.1304861)
Supplement: Supplementary file 2 [file Data_Sheet_1.docx]

**Supplementary File 1. Program to Lower Unwanted Media Screen (PLUMS) Intervention**

PLUMS intervention contains Child and Parent Module and Weekly Intervention Plan as described below:

1. **Child Module**

**Childs Activity journal**

Parents can choose from any of these activities depending upon the interest of the child on a particular day. List of activities is given below:

1. **Customise their favourite book**

Take a coloured sheet and help the child cover or colour or beautify his favourite book.

*What it teaches?*

Improves the child’s creativity and the child would like to read from this book

1. **Plant a seed in a pot**

Buy a small earthen pot for the child and help him/ her to plant a seed. They would love to water it every day and see them grow.

*What it teaches?*

It’s a biology experiment in which the child learns how a seed grows into a sapling

1. **Paint the pot that you bought**

Teach the child to paint the flower pot and they can spend hours together if they start enjoying it

*What it teaches?*

Improves the child’s creativity and the child would feel proud of his/ her art

1. **Make a crown**

Help the child make a crown for themselves. Make a few stars to put it up where everybody can see they have does something good in that day.

*How will this help*

This is a way of inculcating positive habits in the child. The parents need to praise the child when he/ she has been good and punish them when they aren’t behaving well.

1. **Prince/ princess day**

When your child does something good like; listening to instructions, limiting his/ her screen time, doing his/ her homework in time etc. make them wear a crown on their head and play prince or princess for the day.

*Tip*

Do not exaggerate on the rules, or they might not like it. But treat the children like someone special that day

1. **Make a bookmark**

Help the child make a bookmark. Let them design it themselves

*How will this help*

We are trying to inculcate reading habits in the child. When children customise things they like them more and feel responsible.

1. **Make a schedule**

Help the child make up his daily schedule. This should include screen time of 15-20 minutes duration only after they do a good thing. It should have duration of playtime, homework, sleep time and other daily activities.

*How will this help*

Makes the child feel responsible for his actions and empowers him to choose what he likes the most. This inculcates discipline in the child.

1. **Screen free date**

Make sure that you add one day in a week during which the child is allowed to choose from activities that he would like to do but no digital-media gadgets are allowed for that day.

*How will this help*

Children learn to live without media screens and try to find out others ways to play.

1. **Collage**

Make a collage or collection of things the child did in a day/ week. This will be a memoire for the parents.

*How will this help*

The child gets the confidence that he can create something. They will boast this book to everyone who comes home and will be more likely to add pages to this book.

1. **Hand/ finger/ vegetable painting**

Guide the child to paint with hands or feet. Let them get messy. You can also cut vegetables for them to colour and paint on a piece of paper.

*What it teaches?*

It teaches them about colours and art.

1. **Word Board**

A whiteboard/chalkboard easel with magnetic numbers and letters will keep toddlers busy for hours.

*How to Play?*

Use a bulletin board and write down words on strips of paper. The words should be those that your child comes across in everyday life. It will be better if you put a picture next to the word to indicate what it means. For example, truck, car, bus, toy, daddy, mummy, rain, sun, etc.

*What it teaches?*

It improves their reading ability.

1. **Write a letter**

Let them write a letter to people in the family like Grandma, cousins, or a friend.

*What does this teach?*

Children learn to write and express their feelings by writing by their own hand.

1. **Skip Counting**

This activity is one of the simple math activities for 4-5-year-olds.

*How to Play?*

Draw or stick pictures of objects like fruits, cars or animals on cards or notebooks to help your child count. Then skip a few numbers and ask them which one you skipped.

*What it teaches?*

It improves their math skills. This will improve their grades in school and they will enjoy doing it.

1. **Reading a book.**

Read to your children and keep these books where the children can grab them whenever they wish to.

*What it teaches?*

It develops a child’s imagination and they get comfortable in reading. This will eventually improve their grades in school

1. **Write a book.**

Even if your kids can’t write fluently, they can still create stories. You can give them blank notebooks, and let your kids illustrate a story. Later, you can write the words for them as they dictate them to you, or they can figure out the words and write the story on their own. The book can be in any language the child is comfortable in writing and reading,.

*What it teaches?*

This improves their writing skills and improve their vocabulary.

1. **Act out a book.**

Every child has a favourite book? Make them act it out—they may give the story a unique turn, all of their own.

*What is teaches?*

It develops a child’s imagination and they get comfortable in speaking. This will eventually improve their grades in school

#### **Have them make their own cartoon**

Instead of watching cartoons, make your children make their own cartoon figured. Give them a piece of paper and some colours/ crayons. They can make their own hero and a bad guy. When they’re finished making these figures, let them tell you their hero’s story.

*What is teaches?*

It develops a child’s imagination.

1. **Origami**

Origami or paper-folding is an activity that children enjoy a lot.

*How to Make?*

You can use coloured origami paper, craft paper or plain old white sheets coloured for this. Teach your child how to fold paper to make interesting shapes like aeroplanes, rockets, boats, birds, etc.

*What it teaches?*

Improves spatial reasoning

1. **Craft Box**

This is one of the fun activities for 5-year-olds that helps them explore their creativity.

How to Make?

Fill up a craft box with things like pipe cleaners, craft eyes, colourful yarn, safety-scissors, coloured mini-puff balls, ice-cream sticks, felt squares, and tape. Let your child get creative with these. What Does it what *What it teaches?*

Improve the imagination and creativity of the child.

1. **Cardboard boxes.**

Keep the cardboard boxes that you get when you buy something, and just see your child’s imagination: a castle, a spaceship, a sailboat for an adventure to a new world.

*What it teaches?*

Improve the imagination and creativity of the child.

1. **Craft**

Our kids draw or create nearly daily.  If they want to paint, just use that handy old tablecloth again, and spread it on the kitchen floor.

*What it teaches?*

Improve the creativity of the child.

1. **Make an indoor clubhouse.**

Help your child build a fort or cave or den etc. with a blanket and pillows. Get your child started, and see how else they architect a little place of their own.

*What it teaches?*

Improve the creativity of the child.

1. **Perform a puppet show or play.**

Collect old socks and put them over your hand. You can make up your own stories and teach children moral lessons from these puppets. Or kids can create costumes to act out a play, with you as the audience. They can let their stuffed animals star as puppets, and pretend like a movie is going on.

*What it teaches?*

Improve the creativity of the child.

1. **Have an indoor picnic or tea party.**

Lay out your child’s favourite tablecloth on the floor—kids think it’s a big treat to do the everyday chores (like meals) in a special place. You can make some fruit smoothies or shakes or tea, and have a little tea time in cups with saucers, alongside crackers or bread as a treat.

*What it teaches?*

It teaches children to socialise with other kids. They learn how to behave with other children.

1. **Have a playdate.**

Invite your child’s friends over. Or make a neighbourhood kids playdate schedule in which all the parents can fix days when they can look after each other’s kids. This can improve a sense of community in the neighbourhood and the children will make friends.

*What it teaches?*

The child learns to play with other children.

1. **Play restaurant.**

Act like you work in a play hotel and get notepad, pencil, apron, tray, and. Make dishes that the children would like to eat. Sticky notes make good price tags.

*What it does?*

The child would eat in a playful manner

1. **Dance party.**

Even the parents can benefit from such little breaks in the day. Put some peppy music and get dancing. Even 10 minutes of jiving with release some wiggles, and it’s a stress relief for you, too.

*What it does?*

Relaxes the kid and the parent and you can have healthy family time

1. **Play-dough.**

Children absolutely love to make shapes. Spread a tablecloth on the floor, give the child some dull knives, a rolling pin, and some cookie cutters.

*What it teaches?*

Improve the creativity of the child and they can learn shapes.

1. **Squeeze Paint**

Put plain white glue in several empty, squeezable plastic containers and add various watercolours to make bottles of different coloured glue. Now let your child squeeze out various coloured patterns onto white chart paper and let it dry. This will make a colourful display piece once the glue has dried.

*What it teaches?*

It teaches them about colours and art.

1. **Art with Seeds and Grains**

Draw the shape of a flower or an animal like a dinosaur, duck or dog on a sheet of paper. Spread glue inside the shape and ask your child to fill it with seeds and grains to make the picture beautiful. You can use bird seeds, toor dal, masoor dal, moong dal, chana dal etc. to fill the picture.

*What it teaches?*

Kids develop better hand-eye coordination when they pick up little seeds and glue them onto paper.

1. **Parts of a Plant Craft**

Use markers, ice-cream sticks and coloured paper to make a picture of a plant on chart paper. Once this is done, label all the parts such as flower, petal, stem, leaf, root, etc. and teach your child the part of a plant.

*What it Teaches?*

This teaches the kids about the different parts of a plant.

1. **Barbie Dress-up**

Kids love to play with dolls. Create a theme such as a house party or day on the beach and help them dress up their dolls with dresses and accessories to suit the theme. Let them make up stories about each activity that the dolls are engaged in. They can also comb or braid the dolls’ hair and style it with tiny clips that come as doll accessories.

*What it teaches?*

The kids develop their fine motor skills when they work with tiny doll accessories to dress up the dolls.

1. **Sorting Coloured Candy/ vegetables/ fruits**

Give your kids a bowl full of coloured candy like jelly beans or gems. Ask them to sort them colour-wise and arrange them in different cups.

*What it teaches?*

This teaches kids about colours. It also helps them develop their fine motor skills as they use their fingers and hands to sort the little candies.

1. **Art using Leaves and Petals**

First, ask your child to go out to the garden and collect different leaves, petals and tiny sticks. Next, use chart paper to draw an outline of scenery, flowers or a tree. Now spread glue over the outline and stick real petals, tiny branches and leaves to form a beautiful picture.

*What it teaches?*

This helps kids develop both fine and gross motor skills. It also teaches them about nature and plants.

1. **Bubble Art Using Straws**

Fill several cups with mild soap solution and add drops of different food colour to it to make colourful liquid soap. Let your child blow bubbles into the cup with a straw. Make sure the child does not sip or swallow the soap solution. When the bubbles rise to the surface of the cup, place a white card over them. As the bubbles burst, they leave a colourful imprint on the card, making beautiful patterns.

*What it teaches?*

This helps kids use the muscles of their hands, fingers and lip, thus helping them develop fine motor skills.

1. **Moving objects**

Get them vehicles of various types, like cars, trains, trucks, diggers, buses, auto rickshaws, etc. Let them play with the toys.

*What it teaches?*

This teaches kids about various means of transport and the types and uses of different vehicles.

1. **Animal habitats**

Collect plastic animal toys like bears, apes, lions, giraffes, polar bears etc. Help your child use cotton and thermocol balls to make an Arctic habitat covered with snow and icebergs. You can put toys such as polar bears and arctic foxes in the habitat and teach your kids about how these animals live in such places. Similarly, you can make an African or Amazonian jungle.

*What it teaches?*

This teaches kids about animals and their habitats.

1. **Study of Colours**

Cover one or two colours at a time. If you are studying red, collect various things that are red, like a picture of a ladybug, a strawberry, etc. teach your child about that colour.

*What it teaches?*

This teaches kids about colours in nature.

1. **Story Time**

This is one of the best quiet time activities for 5-year-olds.

*What to Do?*

Use different voices, impressions and facial expressions to make the story more fun and animated.

*What it teaches?*

This activity helps you bond better with your child and teaches a love of reading.

1. **Building Blocks**

Give your child legos or building blocks and ask her to make different constructs with them.

*What it teaches?*

This can teach kids about the stability and balance of a construct and help them develop better hand-eye coordination.

1. **Sand Pit**

Let your child play in the sand for castle building and let them play in a sand pit with other children, building sand castles and shapes.

*What Does it Teach*

It teaches kids about the feel and texture of dry and wet sand and also about how to share things and play well with others.

1. **Running through Sprinklers**

Turn on the garden sprinklers and let your child run through them, getting wet.

*What it teaches*

It teaches kids about water and balance. It also is immensely amusing for the children.

1. **Gardening**

Get your child to help you with weeding the garden or planting a sapling or watering.

*What it teaches?*

It teaches kids about plants, soil and how plants grow.

1. **Play Park**

Take your kids out to a play park and let them play with other children.

*What it teaches?*

It teaches kids about how to behave with peers in a social setting and teaches them to get along with other kids of similar age.

1. **Household chores**

And hang up their clothes. Put all the dirty stuff in the laundry. pick up their towel, their pajamas, put the toothpaste cap back on etc

*What it teaches?*

It gives them a sense of responsibility and well you work becomes easy

1. **Let them help make their own meals.**

Kids like to know they’re making some decisions, so if they get to be a part of what they are eating at school the following day.

*What it does?*

it’ll make them feel important.

1. **Rearrange the bedroom.**

If your child is old enough to safely move small furniture around, let him explore his creative side and rearrange his bedroom.  Depending on the result, it could be an temporary set up or a new permanent look.

*What it does?*

it’ll make them feel responsible.

#### **Give them an important mission**

Give your child a task, and make it a really big deal. Tell them they need to draw a picture for Dada, or that they need to make a fort for Grandma. If they think it’s an important job, they won’t complain about working on it independently.

*What it does?*

It will make them feel important.

#### **Generate an idea box**

Brainstorm ideas with your children about what they can do to overcome boredom. Write down their suggestions, and put them in an empty box. Then, the next time they’re bored, have them pick out one of their own suggestions.

*What it does?*

Given that it was their idea, they’ll be more willing to actually do it.

1. **Offer creative toys**

Any toy that lets a child create is sure to keep them distracted for a long time. Invest in Legos, puzzles, etc.

*What it does?*

They’ll build up their spatial reasoning, too.

#### **Design a treasure hunt**

Hide something like a coin or a sticker somewhere in the house. Give your kids a clue, and let them run wild trying to find it. If you make it a bit tricky to find,

*What it does?*

It can build up their resilience and their ability to find things without begging for your help.

#### **Let them play outside**

Don’t forget how your parents kept you busy. Just give your child a ball and a stick, and let them run wild. If you’re worried about their safety, just keep them in sight of the window.

*What it does?*

It’s important for the child’s physical and mental health

#### **Play with locks and bolts**

Hand your child a lock and a key or a nut and bolt and let them play with it. Young kids, especially, will be mesmerized by the act of unlocking something. Give them a mixed bag, and see if they can figure out which lock goes with which key.

*What it does?*

they’ll develop their motor skills

#### **Have messy mixing time**

Set up a big tarp on the floor, and give your child some bowls and things from the kitchen they can mix together. Let them go wild. Your child will be so excited that they get to be messy indoors that they’ll be willing to play without your constant attention.

*What it does?*

It’s important for the child’s physical and mental health

#### **Play verbal games**

If you have the energy to multitask, you can keep your child entertained while still getting things done. Take turns naming an animal for each letter of the alphabet, or play 20 Questions. You can cook, clean, and get ready while quizzing your child.

*What it does?*

Helps children memorise without getting bored.

#### **Create a scavenger hunt**

You don’t need to make a whole list – just say, “Find me something that starts with the letter B,” and let them run around the house searching. To keep your kids motivated, you might reward them by letting them choose a dish for dinner once they’ve found 10 things.

*What it does?*

It can build up their resilience and their ability to find things without begging for your help.

#### **Suggest a science experiment**

Teach or show them something about an object or their surroundings and let them explore it. That might mean giving them a magnet and telling them to see what sticks, or giving them a bowl of water and prompting them to see what will float and what will sink.

*What it does?*

Helps children learn without getting bored.

#### **Give them a new tool**

Especially for young children, just handing a child a new tool can keep them occupied for a pretty long time. Give your kids a flashlight, a combination lock, or a magnifying glass, and let them figure out how it works for themselves.

*What it does?*

Helps children learn without getting bored.

1. **Playing with the pets**

They can play fetch with the dog, or fashion an indoor dog sled (wherein the dog drags them in office chairs around the house) which can go on for a while.

1. **Wait them out**

If all else fails, just wait them out. If you can put up with 20 minutes of your kids whining about you turning off the TV, they’ll eventually find something else to do.

1. **Parents Module**

**Child Milestone Tips for the parents**

I am 2 years old

- I like to copy everyone
- I love playing with other children
- I can do things alone
- I don’t like instructions

Language: I am able to

- Speak small (2-4 words) sentences
- Locate my body parts
- Repeat what I hear
- Recognize similar objects

Learning: I am able to

- Find hidden objects
- Recognize shapes and colors
- Build blocks
- Follow instructions
- Learn songs/ rhymes/ stories
- Name items in a picture
- Turn book pages with help
- Make or copy straight lines/ circles

Activity: I am able to

- Kick/ throw ball
- Run/ jump sometimes
- Climb up and down the stairs/ furniture
- Stand on one foot

Parenting tips:

- Should encourage the child to help with simple house-hold chores
- Praise the child for good things and limit the child’s bad habits.
- Don’t correct the child when he tries to speak, rather you should give an example.
- Encourage the child to say the name of things instead of pointing at them.
- Play with the child at home with toys, puzzles, colors, blocks etc.

I am 3 years old

- I like to copy everyone
- I get emotional when I see someone crying
- I know all things that are mine or daddy’s or mummy’s
- I get upset with big changes in my daily routine
- I can dress up myself
- I love to show a lot of emotions

Language: I am able to

- Name most of the things/ people that I’ve seen before
- Say my name, age and whether I’m a boy or a girl
- Talk to strangers
- Talk in full sentences

Learning: I am able to

- Play with moving toys, dolls, animals and people
- Solve puzzles (3-4 piece)
- Draw a circle
- Play with blocks
- Can open jars, doors etc.
- Turn book pages myself

Activity: I am able to

- Run/ jump easily
- Climb up and down the stairs/ furniture very well
- Pedal my tricycle

Parenting tips:

- Encourage your child to play with other children
- Help your child solve their daily problems
- Talk to your child about his emotions
- Set rules and limits for your child but give instructions in 2-3 steps
- Go outside with your child
- Play counting games, matching objects or activity books with your child.


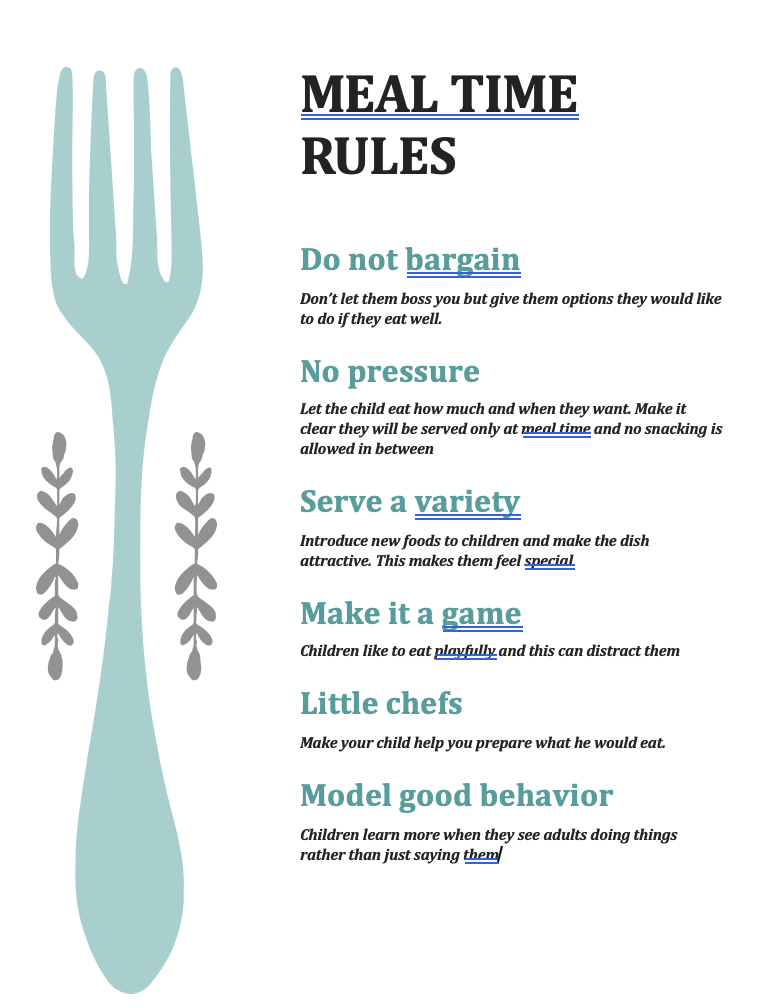

**Weekly Plan:**

| **Weekly calendar of activities for the parents and children** | | | | | | |
| --- | --- | --- | --- | --- | --- | --- |
| **Week and Theme** | **Parent module**  **Session** | **Time required** | **Weekly assessment** | **Child Module**  **Activities** | **Time required** | **Weekly assessment** |
| **Week 1**  **Growing up** | Excessive screen-time and development milestones of children | 30 minutes | Compliance checklist | Activity booklet, list of optional activities and comic book | 30 - 60 minutes | Activity booklet |
| **Week 2**  **Screens away** | Screen-time rules at home | 30 minutes | Compliance checklist | Activity booklet and list of optional activities | 30 - 60 minutes | Activity booklet |
| **Week 3**  **Sound sleeper** | Sleep and digital media | 30 minutes | Compliance checklist | Activity booklet, list of optional activities | 30 - 60 minutes | Activity booklet |
| **Week 4**  **Home sweet home** | Home media environment | 30 minutes | Compliance checklist | Activity booklet and list of optional activities | 30 - 60 minutes | Activity booklet |
| **Week 5**  **Hungry** | Meal time and digital media gadgets | 30 minutes | Compliance checklist | Activity booklet and list of optional activities | 30 - 60 minutes | Activity booklet |
| **Week 6**  **Chatter box** | Effective Communication within family | 30 minutes | Compliance checklist | Activity booklet and list of optional activities | 30 - 60 minutes | Activity booklet |
| **Week 7**  **Play together** | Education in positive experiences in a community setting | 30 minutes | Compliance checklist | Activity booklet and list of optional activities | 30 - 60 minutes | Activity booklet |
| **Week 8**  **Happy baby** | Positive reinforcement and counseling | 30 minutes | Compliance checklist | Activity booklet and list of optional activities | 30 - 60 minutes | Activity booklet |
